# Outstanding Approach to Enhance the Safety of Ready-to-Eat Rice and Extend the Refrigerated Preservation

**DOI:** 10.3390/foods11131928

**Published:** 2022-06-28

**Authors:** Najla A. Albaridi, Ahmed Noah Badr, Hatem Salama Ali, Mohamed Gamal Shehata

**Affiliations:** 1 Nutrition and Food Science, Department of Physical Sport Science, College of Education, Princess Nourah bint Abdulrahman University, P.O. Box 84428, Riyadh 11671, Saudi Arabia; naalbaridi@pnu.edu.sa; 2 Department of Food Toxicology and Contaminants, National Research Centre, Dokki, Cairo 12622, Egypt; 3 Department of Food Technology, National Research Centre, Dokki, Cairo 12622, Egypt; hatem.owyean1@gmail.com; 4 Department of Food Technology, Arid Lands Cultivation Research Institute, City of Scientific-Research and Technological Applications (SRTA-City), New Borg El-Arab 21934, Egypt; gamalsng@gmail.com; 5 Food Research Section, R&D Division, Abu Dhabi Agriculture and Food Safety Authority (ADAFSA), P.O. Box 52150, Abu Dhabi, United Arab Emirates

**Keywords:** antifungal, aromatic and water extract, encapsulation, microbial inhibition, ready-to-eat rice, spices mixture, shelflife extended

## Abstract

Rice is a broad-spectrum meal consumed annually in large amounts. Ready-to-eat rice is a member of dishes with a high risk of contamination. The present study aimed to increase the safety and shelflife of ready-to-eat rice during temporary storage. To prepare a mixture for extraction, three spices were chosen ginger: thyme:coriander (1:2:1). Two types of extract were prepared, aromatic and water extracts. The bioactive aromatic extract was preserved by encapsulation using chitosan nanoparticle preparation, while water extracts were prepared by warm diffusion. The aromatic extract possessed volatiles with antimicrobial features, including α-pinene, cymene, camphor, 1, 8 cineol, and limonene. The results expressed the extracts’ better antifungal and antibacterial effect, with a distinguishing aromatic one. Water extract was recorded as being rich in phenolic and flavonoids, like Salysilic, p-hydroxybenzoic acid, ferulic, Luteolin 7 glucoside, and quercitin. These molecules play functionality for microbial inhibition in the simulated media. Ready-to-eat rice shelflife was extended by applying the aromatic extract of the encapsulated mixture at the late stage of cooking and before packaging. It can preserve the samples for up to five days at room temperature and up to eight days of refrigerator storage (8 °C). However, water extract had lower activity as antibacterial and antifungal than the aromatic one. Again, water extract activity reduces fungal citrinin secretion by low efficiency more than the aromatic extract. These results recommended the addition of aromatic extract to the ready-to-eat rice meals as a final additive just before packaging.

## 1. Introduction

Rice is the world’s second most significant crop and a staple diet for half of the world’s population. It is cultivated in more than a hundred countries, with a massive worldwide output measured in millions of tons [1]. It is one of the most significant crops in Saudi Arabia and Egypt, with a high consumption rate. Rice cultivation is essential to the government’s effort to combat food shortages and increase domestic consumption and export self-sufficiency. It is a healthful cereal grain that is appropriate for various nutritional demands. It is composed chiefly of carbohydrates, protein, and low fats, similar to wheat. Several types of cooked rice are regarded as the main dish of the noon meal, whether with or without meat. Kapsa is a classic sort of spiced cooked rice dish.

Flavored or spiced cook-rice are meals consumed in food habits of a particular population like South-east Asia and traditional Nomad meals. The use of spices is signifying. The spicy rice meals and the type of spices constitute the desired taste in the food products [2], and its components of biologically active substances may be lost by heat treatment. There is a request to valorize these molecules’ functionality in food processing and preservation, considering the functionality of these substances and their participation by a critical role in the food preservation processes [3]. Encapsulation of spices extract or the delay of their addition could protect their bioactive molecule contents and increase their functionality in cooked-rice preservation [4,5]. Otherwise, the methodology of spice extraction could also affect the extract’s molecule concentrations. One more methodology for enriching the applied extract’s bioactive components is its formulation as a mixture of more than one spice type. Generally, ginger, cumin, coriander, pepper, oregano, and thyme are the spices used in Kapsa preparation [6].

Bacterial count in prepared food is critical in determining food quality and safety [7]. The microbiological quality of food reflects the quantity of microbial contaminants, with a high degree of contamination suggesting a lack of food quality and a higher risk of disease transmission. It also displays the cleanliness used by food handlers throughout the meal preparation process [8]. Pathogenic bacterial strains, *coliforms*, *Bacilluscereus*, and *Salmonella* presence in food items have been detected and evaluated to ensure hygienic-sanitary quality during handling and storage circumstances [9]. The strain of *B. cereusis* deemed to be the main hazard that threatens ready-to-eat meals, including cooked rice [10]. At the same time, *Bacillus* and *Micrococcus* were prevalent pathogens associated with grain contamination, particularly rice [1]. The bacterial strain of *B. cereus* develops rapidly and generates toxins on boiled rice and other carbohydrate-rich meals kept at room temperature [11]. There is a growing global need for high-quality, safe food free of chemical, physical, and microbial contamination.

On the other hand, fungal contamination is a second microbial contamination type that threatens ready-to-eat rice [12]. Contrary to bacteria, fungi can grow on cooked rice under refrigeration conditions, with a possibility of toxin production [13]. *Aspergillus* and *Penicillium* strains are the primary fungi genera correlated to rice [14]. Aspergilli genera contain *A. flavus* and *A. parasiticus*, aflatoxigenic fungi producing aflatoxins. Aflatoxins are included by aflatoxin B_1_ (AFB_1_), aflatoxin B_2_ (AFB_2_), aflatoxin G_1_ (AFG_1_), and aflatoxin G_2_ (AFG_2_) that are hazardous types of mycotoxins and mainly considered pre-carcinogens [15,16]. The second genera belong to the *Penicillium* genera, which are known to produce several mycotoxins, including penicillic acid, Ochratoxin A, and citrinin [17]. These mycotoxins are causes of several public health issues. Thus, contamination of ready-to-eat meals, especially cooked rice, with fungi is a problem of considerable proportions, whether of fungal contamination as microorganisms or over their secondary metabolites known as mycotoxins.

Modern strategies are requested as intelligent solutions for the contamination in ready-to-eat food types, including cooked rice. Therefore, the investigation aimed to evaluate the impact of spices mixture extracts to increase the stability and shelf life extension of ready-to-eat food. Regarding their preservative effect, a comparison was made between two extraction models (aromatic and water extract) for the mixture of thyme, ginger, and coriander (2:1:1). The previous work pointed out the individual uses of thyme, ginger, or coriander as a flavoring agent and served in preservation. The present study utilizes the spice mixture extract containing bioactive molecules with antibacterial, antifungal, and antimicrobial properties to increase cooked rice’s shelf life and safe characteristic stability.

## 2. Materials and Methods

### 2.1. Sample Collection

Sample rice from five different sources was collected to evaluate their initial load of microorganisms. The samples focused on assessing the commercial rice handled in the Saudi markets. These were commercial Egyptian rice (Giza 179) and Indian rice (white basmati 370, Pusa basmati 1121, and golden-sella basmati 1121). The samples were purchased from a herbal store, sourced from an identical farm in Ismailia, Egypt. Scientific experts identified and authenticated the plant varieties. The plant samples were collected from a certified herbal store, and experts recognized seeds and plant parts. After washing well with distilled water, samples were dried in the shade and ground to pass through a 40 mesh sieve.

### 2.2. Microorganisms and Chemicals

The strains *Bacillus cereus* EMCC 1080, *Staphylococcus aureus* ATCC 13565, *Micrococcus luteus* ATCC 15176, *Escherichia coli* ATCC 51659, *Salmonella typhi* ATCC 15566, and *Campylobacter jejuni* ATCC 33560 were utilized for antibacterial assay and obtained from the DSMZ microbial collection (Leibniz Institute DSMZ-German Collection of Microorganisms and Cell Cultures, Braunschweig, Germany). It was reactivated (24 h/37 °C) using nutrient agar slants. While toxigenic fungal strains of *Aspergillus flavus* ITEM 698; *A. parasiticus* ITEM 11; *A. niger* ITEM 3856; *Penicillium chrysogenum* ATCC 10106, and *P. verrucosum* NRRL 965 were utilized for antifungal assay. These strains were obtained from the microbial culture collection of the Institute of Sciences of Food Production, ISPA (Bari, Italy).

### 2.3. Selection of Spices for Rice Preservation

Based on common spices used in rice cooking, the authors create a bioactive-molecules mixture extracted from 3 sources ginger, coriander, and thyme. The presence of bacteria or fungi is expected to cause microbial spoilage for the products during the storage conditions. In another respect, the individual extract of coriander, thyme, or ginger in recorded literature has possessed antimicrobial potency. The application of the mixture target the preservative action properties during cooked rice’s plain and cooled storage. This mixture ameliorates ready-to-eat rice’s shelf life as natural, healthy, and chemical-free preservation.

### 2.4. Extraction of Aromatic Compounds

Seeds were treated with sodium hypochlorite (3%/2 min) for fungal sterilization, followed by washing (3 times using deionized water) and air-drying before extraction. According to the methodology described by British Pharmacopeia described by Jalali et al. [18], the aromatic components were extracted using a two-liter of Clevenger-type hydro distillation unit. The hydrodistillation was carried out (4 h) in a device until the contained volatility was exhausted. The device was charged with 200 g of ginger: thyme: and coriander (1:2:1); the ratio was chosen according to the preliminary studies for the antimicrobial effect. An aromatic extract (ArE) will be gained from this mixture and used for further studies.

### 2.5. Preparation of Water Extract (W.A.)

The water extract (W.A.) for the same matrix of the previous mixture (ginger:thyme:coriander; 1:2:1) was performed using a hot water diffusion system described by Abdel-Salam et al. [19]. Briefly, cheesecloth containing a 200 g mixture matrix was submerged in a horizontal-double jacket basin with conducted pump tubes. A continuous system using a peristaltic pump (Model S300-12B, Baoding Ditron Electronic Technology Co., Baoding, China) at 50 °C/4 h was applied. The obtained extract was concentrated by a rotary evaporator (Heidolph, HeiVAP, GmbH, Landsberger, Germany), then kept in an amber bottle.

### 2.6. Determination of Bacterial and Fungal Count

Collected rice samples were inspected and evaluated for their initial load of microorganisms. The total bacterial count of samples was determined according to Tahir et al. [20] methodology, with modification. Briefly, 5 g rice was added to a sterile flask containing sterile normal saline (10 mL), and this flask was considered the stock for the following 4-fold dilutions. Each dilution was utilized to inoculate the plate count agar media in triplicates, where these plates were incubated at 37 °C/ 24 h before results were calculated. Thetested samples expressed the bacterial growth in a CFU/mL.

Rice samples were tested for their loading of toxigenic fungi as the methodology described by Makun et al. (2007). Ten grams of rice were sterilized with sodium hypochlorite solution (3%) and then washed with sterile distilled water (in 3 replicates). Ten grains were embedded randomly in the Petri-dishes of potato dextrose agar (PDA) supported by chloramphenicol (500 mg /liter) and then were incubated (28 °C/4 days). Fungi from plated grains were replanted using the single spore technique. T then transferred to PDA slant media bottles and fresh PDA in Petri-dishes for morphological identification; isolates were identified using the description of Alsohaili and Bani-Hasan [21].

### 2.7. Determination of Aromatic Compounds by GC-MS

The aromatic compounds were evaluated by an Agilent G.C. 7890A with a split injector port set to 200 °C and a scan ratio (200:1). Agilent G.C. 7890A evaluated aromatic compounds with a split injector port set to 200 °C and a scan ratio (200:1). An auto-sampler (Agilent 7697A) was employed for performing the static headspace sampling. The vials were equilibrated (70 °C/10 min) before sampling with the loop (85 °C). Separation was achieved using a DB-624 capillary column (30 m 0.25 mm I.D. 1.4-m film thickness) with a continuous helium carrier flow (1 mL/min). The starting oven temperature was 45 °C for 3 min, followed by a 3 min ramp to 240 °C/25 °C/min rate. The Agilent detector 5975 MSD was utilized to identify and quantify volatile chemicals in the scan range (29–250 *m*/*z*; 6.1 scans/s). TheMassHunter program (B.09.00) constructed all calibration curves and sample concentrations.

### 2.8. Encapsulation of Aromatic Extract

The chitosan nanoparticles were performed in the same methodology described by Jarudilokkul et al. (2011) with few modifications [22]. About 1.5 mg/mL, chitosan solution was prepared by dispersing 22.5 mg of chitosan (90-degree de-acetylations) into acetic acid (2.25 mg/mL). The extracted molecules of essentials (of the previous mixture) were dissolved in Tween 60 using a magnetic stirrer (37 °C/2 h), followed by dropwise addition during the stirring to encapsulate using chitosan solution (50 mL). The final solution was mixed with 20 mL of tri-polyphosphate solution (1 mg/mL). The solution was stirred (25 °C/30 min) to form an opalescent suspension, and then centrifugation (10,000× *g*/4 °C/30 min) separated the chitosan nanoparticles.

### 2.9. Characterization of Encapsulated Aromatic Extract

The characterization of chitosan nanoparticles was estimated by particle size, zeta potential, and polydispersity index (PDI) utilizing nano-ZS, Zeta-sizer, Malvern Instruments Ltd., U.K. equipment. The investigated materials were homogenized in ultrapure water (1 mg/mL) before being passed through a filter syringe (0.45-m), which improved the removal of insoluble particles.

### 2.10. Determination of the WA-Bioactive Molecules

The phenolic composition of the applicable mixed extract was determined utilizing RP-HPLC-PDA as the procedures provided by Del Pozo-Insfran et al. [23]. Moreover, following the conditions and elution gradients modified as stated by Aguilar and Hernández-Brenes [24]. In brief, the mobile phase consisting of water (phase A) and methanol (60%; phase B) was used, where the pH 2.4 was adjusted using orthophosphoric acid. In methanol HPLC grade, samples were diluted 1:1 with 1.2 M HCl solution and filtered through 0.45 m PTFE membranes (Gelman, Ann Arbor, MI, USA). The peaks of the results were identified using comparisons to authentic standards and retention times. The chromatograms were investigated at three different wavelengths (280; 335; 380) based on their chemical nature.

### 2.11. Determination of Antibacterial and Antifungal Properties

The antibacterial assay was accomplished using the diffusion assay according to the methodology described by Abdel-Razek et al. [25]. Otherwise, the antifungal assay was conducted against the tested extracts to evaluate their activities [26]. The result of inhibition was expressed as a millimeter diameter of zone-inhibition (mm).

### 2.12. Antiaflatoxigenic Properties in Simulated Media

The obtained aromatic and water extracts were applied in fungal media of toxigenic strains to evaluate their efficacy in limiting toxin production. Liquid media of yeast extract sucrose, which contains the spores of *A. flavus* or *P. chrysogenum*, were individually inoculated by the extract types. The experiment was classified into flask groups of six groups G1:flasks contain spores of *A. flavus*; G2:flasks contain spores of *A. flavus* + the W.A.; G3:flasks contain spores of *A. flavus* + ArE; G4: flasks contain spores of *P. chrysogenum*; G5: flasks contain spores of *P. chrysogenum* + the W.A.; G6:flasks contain spores of *P. chrysogenum* + the ArE. The flasks were tested for fungal growth inhibition after incubation for four days (22 °C), while the toxin reduction was evaluated after 12 days of incubation (28 °C). The methodology of Shehata et al. [27] had been applied to calculate the reduction in mycelial fungal growth as an inhibition ratio of the control flasks. Similarly, the reduction in toxin secretion was calculated compared to the control flasks.

### 2.13. Mycotoxin Determination in Simulated Media

The examined flasks were filtered using filter paper (Whatman No. 1) to collect the media extract containing the suggested mycotoxin. The filtrate of *A. flavus* flasks was evaluated to measure its aflatoxins content, while the filtrate of *P. chrysogenum* flasks was considered to estimate its citrinin content. The aflatoxin content was determined according to the methodology and the conditions described by Shehata et al. [27]. The HPTLC was utilized to evaluate the reduction in citrinin mycotoxin in media [28]. In brief, the sample was run on the plates and placed in a chamber previously saturated with the mobile phase. The mobile phase was used in a linear ascending development of toluene, ethyl acetate, and formic acid in the ratios of 3:2:1 (*v*/*v*). The running system condition was modified, and the scanning samples were taken under the same conditions [29]. As a reference for the calibration test, a 1 µL citrinin injection was administered.

### 2.14. Simulated Experiment for Bacterial Contamination in Ready-to-Eat Rice

Rice samples of ready-to-eat meals were prepared using a commercial rice-cooking machine (CUCKOO CR-0631F, Cuckoo Electronics Co., South Korea), which has a capacity of 6 cups of un-cocked rice, supplemented with a standard cup and a plastic spoon. The machine pan was filled with rice (300 g) and water saline (450 mL; 2% salt) for each control treatment, nano-ArE, and W.A treatments. Samples were cooked for 35 min for the control; however, for extract treatments, it was cooked firstly for 30 min, then a targeted extract for each treatment was added (1 mg dissolved in 25 mL water) before finalizing the cooking time. The extract types’ applied concentration was chosen according to the preliminary experiment to examine the minimal inhibition concentration impact.

The rice of each treatment was transferred to an eco-friendly disposable PET round bowl with a sealed cap. The bowls were sterilized with antibacterial dish soap followed by peracetic acid treatment soaking (5%; 3 min), dried using sterilized tissues, and finally, placed for 10 min under UV-lamp exposure. The sterilized bowls were used for dividing rice treatments into groups of treatments. Cooked rice bowls were divided into two sets (inoculated and non-inoculated). The inoculated set bowls were treated using *B. cereus* (0.24 × 10^2^), prepared using serial dilutions, and adjusted using Bürker Counting Chambers. At the same time, non-inoculated bowls had no additives except the applied extract type. Samples were stored at room temperature (25 °C) or refrigerated (8 °C) during the storage time under two different conditions. The shelflife simulated experimental was evaluated as the same methodology described by Badr et al. [30]. The differentiation of bacterial count content will reflect the efficacy of the applied extract in protecting the ready-to-eat rice samples and their impact on the shelflife.

Sensory evaluation of cooked rice samples was evaluated. The expert team assessed the samples for taste, color, aroma, consistency, texture, and overall acceptability. The evaluation degree was expressed from 10 points, where the more point recommended more acceptability.

### 2.15. Statistical Analysis

The results were conducted in triplicates and expressed as means ± standard deviation (S.D.). The statistical data analyses were performed using the ANOVA-one-way test, and the graphs were represented using Graph Pad Prism 7 (Graph Pad Software Inc., San Diego, CA, USA).

## 3. Results

### 3.1. Normal Contamination of Rice Samples

The functional count of bacteria and fungi reflected the normal contamination of raw-rice samples. The collected rice samples’ fungal contamination was recorded as 2.7 × 10^2^ CFU/mL, and 0.8 × 10^2^ CFU/mL for Giza 179 and white basmati, respectively. The collected samples recorded the other two varieties of Pusa basmati 1121 and golden-sella basmati 1121 as free of fungal contamination. Regarding the bacterial load, the rice variety of Giza 179 was recorded to have 2.1 × 10^2^ CFU/mL; white basmati 370 load was 1.8 × 10^2^ CFU/mL; however, bacterial load for Pusa basmati 1121 and golden-sella basmati 1121 were recorded at 1.4 × 10^2^ CFU/mL and 0.9 × 10^2^ CFU/mL, respectively. In this concern, white basmati was chosen as an intermediate variety with moderate contamination to be applied in further investigations regarding the safety application and shelflife extension applications.

### 3.2. Determination of Aromatic Molecules

The aromatic extract analysis results represent a variation of active molecules known to possess a functionality for antimicrobial impact. Major components in the aromatic extract were recorded for thymol, followed by linalool, thymyl acetate, α-zingiberene, and geraniol (Table 1).

However, other compounds like α-pinene, cymene, camphor, 1, 8 cineol, and limonene possess antifungal activity besides the antibacterial effect recorded in considerable amounts. The result also points out the existence of citrus compounds, including citral, neral, geraniol, and geranyl acetate. These compounds possess antibacterial impact, particularly against pathogenic bacteria.

### 3.3. Characterization of Encapsulated Aromatic Extract

The oil measurements that were encapsulated using chitosan in the form of fine suspended particles showed good properties in terms of grain size, zeta value, and PDI value. The size of the particles when using the electron microscope ranged between 54- and 97 nm. In contrast, the average size of the particles estimated using the Malvern size analyzer was an average of 91.74 ± 3.41. The Zeta potential value of the solution used was 20.11 ± 2.91, while the poly dispersing index (PDI) value was 0.254 ± 0.004 for the particle distribution in the prepared emulsion.

### 3.4. Determination of the WA-Bioactive Molecules

The analysis for the water extract mixture of ginger:thyme:coriander (1:2:1) was reflected by a valuable content of phenolic compounds. Phenolic acids and flavonoid compounds represented these compounds. Eighteen phenolic acids were analyzed, and sinapic acids were recorded as not detected (N.D.). Regarding the flavonoids, two compounds of Isorhamnetin-3-o-rutinoside and Chrysin were recorded as N.D. The mixture extract was distinguished by a high content of phenolic acids like Salicylic, Ellagic, Caffeine, p-hydroxybenzoic acid, and Ferulic acids. However, Hesperidin and Luteolin 7 glucoside were the main flavonoids that characterize the water extract of the mixture.

### 3.5. Determination of Antibacterial and Antifungal Activity

**Figure 1:**
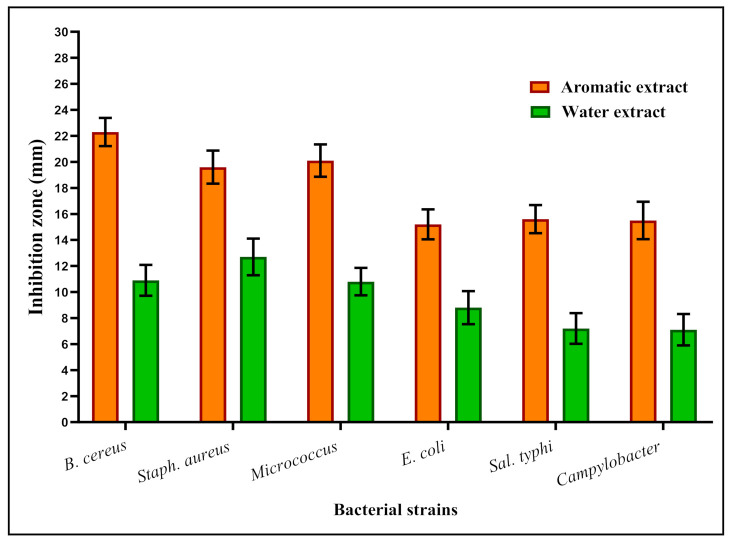
Antibacterial of a mixture (ginger:thyme:coriander) extract types against pathogenic bacterial strains. Results were expressed as means± S.D. (n = 3), inhibition zone diameter represented in millimeters. The aromatic extract was applied in the nano-form of chitosan nanoparticles.

The aromatic and water extracts were evaluated for their antibacterial activity against six strains related to foodborne illness. The results reflect the efficacy of aromatic extract to possess inhibition zones more than their correspondence ones resulting from the water extract (Figure 1).

It is worth mentioning that aromatic extract recorded a valuable impact against the most hazardous bacterial strain, *B. cereus*, which is known to be the main factor of spoilage in ready-to-eat rice. The inhibition impact of the water extract mixture against the same strain was also recorded, but was still lower than the aromatic one effect (Figure 1). Other bacterial strains of *Campylobacter jujini* and *Staphylococcus aureus*, which are known to contaminate cooked rice, were affected. The presence of aromatic extract inhibited their growth more than the water extract application.

**Figure 2:**
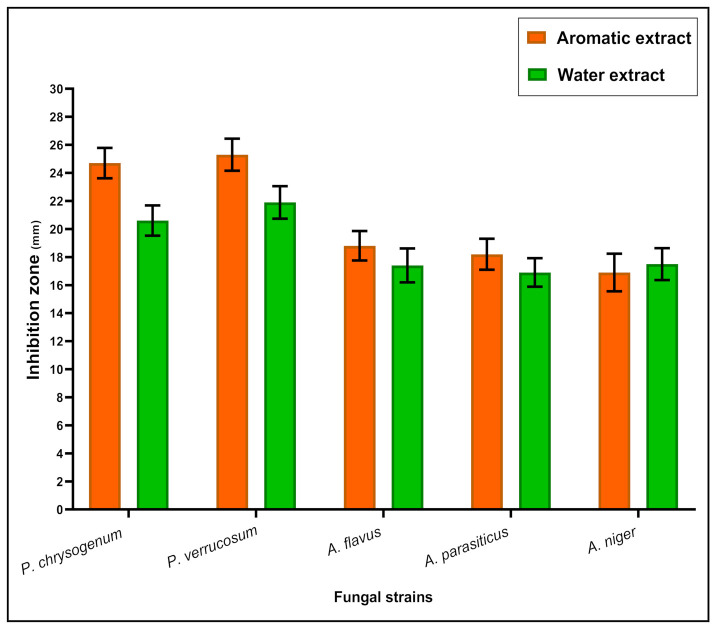
Antifungal and anti-mycotoxigenic effect of a mixture (ginger:thyme:coriander) extract types against pathogenic bacterial strains. Results were expressed as means± S.D. (n = 3), inhibition zone diameter represented in millimeters; The aromatic extract was applied in the nano-form of chitosan nanoparticles.

The effect of aromatic and water extracts against the mycelia growth of toxigenic fungi manifested a closer impact on the two types of extracts (Figure 2). The strains related to *Penicillium* fungi were recorded with more sensitivity for applying the extract than those associated with *Aspergillus* fungi. These results seemed to be more suitable for the aim of this work, which targeted the safety of ready-to-eat rice.

### 3.6. Antiaflatoxigenic Properties in Simulated Media

The first part of the simulated experiment was applied to evaluate the effect of water and aromatic extracts of the mixture (ginger:thyme:coriander) against an aflatoxin-producing strain of fungi (*A. flavus* ITEM 698). The results expressed in Table 2 reflect the inhibition ratio of the aromatic extract (51.59%) by its application in the liquid media of fungal growth. This ratio was recorded at 62.01% by applying the water extract to the fungal growth media.

A reduction in the aflatoxins (AFB_1_, AFB_2_, AFG_1_, and AFG_2_) was recorded for the content of liquid filtrate media. The reduction ratio of total aflatoxins (AFs) for the water extract application (67.11%) was more than the percentage recorded by aromatic extract application (42.82%) in fungal growth media.

### 3.7. Anti-Penicillium Properties and Toxin Reduction

**Figure 3:**
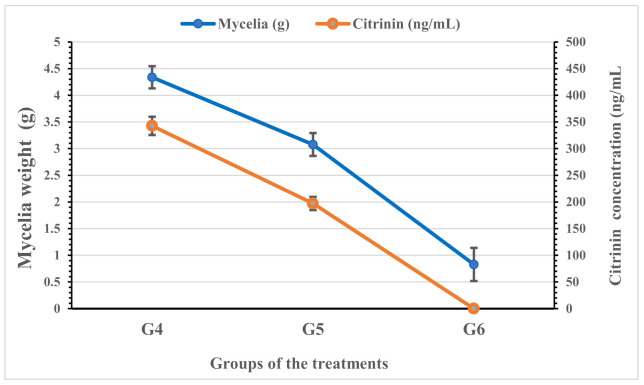
Anti-*Penicillium* properties of applied extracts (water and aromatic) in simulated media. The results are expressed as means ± SD (n = 3); WA: water extract; ArE: aromatic extract. The aromatic extract was applied in the nano-form of chitosan nanoparticles. *P. chrysogenum* is known to produce citrinin toxin. G4: flasks contain *P. chrysogenum*; G5: flasks contain *P. chrysogenum* + the W.A.; G6: flasks contain *P. chrysogenum* + ArE.

The second part of the simulated experiment was applied to evaluate the effect of water and aromatic extracts of the mixture (ginger:thyme:coriander) against a citrinin-producing strain of fungi (P. chrysogenum ATCC 10106). The results expressed in Figure 3 reflect the significance of inhibition for the aromatic extract compared to the impact of the water extract in the liquid media of fungal growth. While applying aromatic extract in the fungal media could limit the toxin secretion, the amount of toxin recorded was not detected. It is worth noticing that, while an excellent ratio did not record the inhibition of fungal growth by water extract application, the toxin limitation was represented in a valuable reduction amount.

### 3.8. Sensory Evaluation of Ready-to-Eat Rice Samples

**Figure 4:**
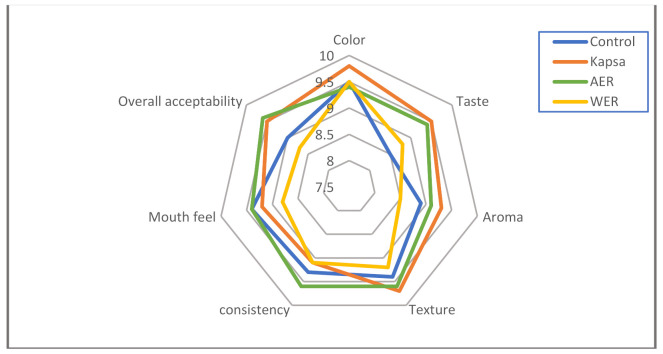
Sensory evaluation of various recipes of cooked rice samples. The control represents normal rice cooking recipe; Kapsa represents the traditional recipe of spice rice, AER: represents the rice treated by aromatic extract of the spice mixture; WER: represents the rice treated by water extract of the spice mixture.

The result for the sensory evaluation of cooked rice samples (control without additives, Kapsa recipe, Rice with aromatic extract, and rice with water extract) reflected the close results between normal Kapsa-rice and aromatic extract rice preparations (Figure 4). The control rice reflects the normal cooking rice without Kapsa or extracts in the recipe.

### 3.9. Simulated Experiment for Bacterial Contamination in Ready-to-Eat Rice

**Figure 5:**
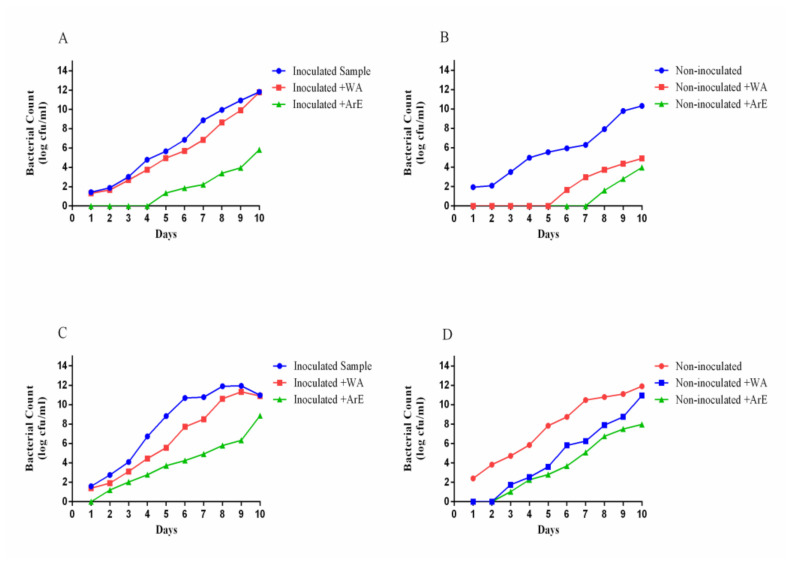
The effect of the mixture (ginger:thyme:coriander) extract types at two storage temperatures, the safety properties of inoculated (*B. cereus*) and non-inoculated samples of ready-to-eat rice. (**A**): effect of aromatic (ArE) and water (W.A.) extracts against inoculated *B. cereus* growth at refrigeration condition of ready-to-eat rice; (**B**): effect of aromatic (ArE) and water (W.A.) extracts against normal bacterial contamination at refrigeration condition of ready-to-eat rice; (**C**): effect of aromatic (ArE) and water (W.A.) extracts against inoculated *B. cereus* growth at a room temperature storage; (**D**): effect of aromatic (ArE) and water (W.A.) extracts against normal bacterial contamination at room temperature storage. The results were expressed in CFU/g for the rice examined samples, the aromatic extract was applied in the nano-form of chitosan nanoparticles.

The samples of ready-to-eat rice, which were treated with the aromatic type of the extract mixture (ginger:thyme:coriander), were recorded as having more safety characteristics for storage and as being less bacterially loaded during the storage period (Figure 5). This property was clear for the count of CFU/g rice calculated at refrigeration (8 °C) or room temperature (25 °C) condition of storage (Figure 4). The result also reflects the significant impact of the water extract application against the contamination of ready-to-eat rice by B. cereus, but less effective than the aromatic extract. The inoculated samples recorded resistance to bacterial contamination up to 4 days of refrigerator storage. At room temperature storage, the aromatic extract treatment of ready-to-eat rice samples had a capacity of up to 2 days of resistance against bacterial growth. Otherwise, the treated water-extract samples were slightly less than the non-treated samples under the same conditions.

Regarding the non-inoculated samples of ready-to-eat rice, the refrigeration condition of storage gives the preference for the aromatic extract to preserve the rice against microbial contamination for up to 8 days, while the resistance property decrease to 5 days in case of the water extract application. It was noticed that the impacts of water and aromatic extracts against the growth of normal flora in the ready-to-eat rice samples were so close (at room temperature storage conditions).

## 4. Discussion

Although flavored and spiced types of cooked rice have broad-spectrum applications and are commonly used in meals of various populations, the application of these spicesmay lose their activity as preservatives regarding their application methodology. Ginger is one of the familiar spices with broad applications. Ginger contains bioactive substances, including zingiberene, gingerols, shogaols, bisabolene, and monoterpenes [31]. Again, the ginger content of active molecules was reported to have antimicrobial and anti-mycotoxigenic impacts by their utilization in food [32]. Coriander is a spice with an antimicrobial effect against a broad spectrum of microorganisms, where various volatile constituents distinguish it [33]. The third kind of spice was thyme, which is utilized in folk medicine besides its food applications. The extract of thyme is rich in volatile and aromatic compounds, and previous research pointed out the functionality of its ingredients as anti-aflatoxigenic and antimycotic substances.

Aromatic molecules and volatile components such as mixture extract as α-pinene, D-limonene, limonene, and cymene are compounds that have antimicrobial effects and are shown in the mixture extract (Table 1). Each of these applied types of spices possesses a group of volatile constituents that distinguish it, and these constituents mainly participate in their bioactivity. At the same time, α-curcumene and α-zingiberene are the main compounds found in ginger volatiles and possess activity as antibacterial and antifungal agents [34,35]; we recorded that this existed in the mixture of the aromatic extract. Thyme extract is distinguished by thymol, thymyl acetate, and geranyl acetate, which are considered volatile constituents [36,37]; they are also present in the prepared mixture of the aromatic extract. Several investigations also referred to the bioactivity of these components and their antimicrobial impact [38,39]. The results represented in Table 1 reflect the rich contents of aromatic mixture extract in terpenes, particularly monoterpenes, which have the activity to act several functions in biological systems. Terpenes are the most abundant category of secondary metabolites in plants, serving basic functions in development, growth, and defense against pathogens [40]. Monoterpenes effectively remedy early and late chronic illnesses [41]. Monoterpenes can prevent cancer types such as liver, kidney, pancreatic, lung, and prostate [42]. According to research, monoterpenes have a variety of therapeutic properties, including antioxidant, antibacterial, antifungal, anti-inflammatory, and anticancer properties [41,42].

Otherwise, phenolic acids and flavonoids reflect the ready water extract from the spice mixture (Table 3). These contents play a significant function in the antifungal and anti-mycotoxigenic activity of the mix of water extract [43]. Phenolic acids such as p-hydroxybenzoic, ferulic, and caffeic were previously reported to have anti-mycotoxigenic and antimycotic effects [44]. Furthermore, flavonoids of luteolin 7 glucoside and hesperidin were represented as principal flavons of the extract. It was reported to possess anti-aflatoxigenic properties with limited impact on mycotoxin secretion by fungi [43,45].

By applying the two types of extracts individually for antibacterial diffusion assay (Figure 1), the results showed an effective impact against pathogenic bacterial strains, particularly B. cereus, which represents the dominant spoilage of cooked rice. Again, it has efficacy in inhibiting the growth of the campylobacter strain known to contaminate ready-to-eat rice meals. The ready-to-eat rice is known to suffer from food spoilage during the storage conditions, including B. cereus, Staphylococcus aureus, and campylobacter strains [1,10]. The impact of the extract on strains known to cause foodborne illness is worth mentioning, including staphylococcus, Salmonella, and E. coli. This impact of antibacterial properties for the extract types is connected to their content of aromatic compounds like terpenes in the aromatic extract [38,40], besides some phenolic compounds recorded in the water extract of the spice mixture that possessed an antibacterial effect [46] and explains the behavior of water extract against applied strains of bacteria.

Moreover, using the antifungal diffusion assay, the results of aromatic and water extracts seemed to be so close to each other against the applied fungal strains (Figure 2). The aromatic extract recorded antibacterial and antifungal properties [47], which recommended it be more effective to achieve preservative properties by its application in ready-to-eat meals. Encapsulation was a recommended technique for increasing the activity of the aromatic extract and protecting its components against spontaneous oxidation or breakdown [48]. The present investigation targeted encapsulating the extract using chitosan nanoparticles, which supports the antibacterial properties and enhances the extract activity [5].

As an application, two simulated experiments were designed to evaluate the efficacy of the extract types, a simulated experiment against toxigenic fungi in liquid media, and the second experiment was conducted in a simulated model of ready-to-eat rice. However, toxigenic fungal strains contaminate cereal grains, including rice [17]; some of these fungal strains are reported to have contaminated the finished products like ready-to-eat rice [10]. The existence of aromatic and volatile components in the simulated media could inhibit microbial contamination [32,42], with or without affecting their secondary metabolites production. On the other hand, phenolic compounds are effective components that act as toxigenic fungi inhibitors [43,49], which could reduce the mycotoxins present in the growth media [50,51]. The experiment, which was conducted in the liquid media of toxigenic fungi strain (*A. flavus* ITEM 698), reflects the efficiency of water extract compared to the aromatic extract as an antimycotic antimycotoxigenic factor. The efficiency of water extract was joined to its content of phenolic and flavonoid compounds [44,52]. The aromatic extract has an anti-*Penicillium* effect, inhibiting *P. chrysogenum* in liquid media.

In the same way, citrinin, considered the related mycotoxin, was reduced by water extract application in the growth media of *P. chrysogenum* fungi and was not detectable by the aromatic extract application (in nano-chitosan form). These results are explained due to the aromatic extract contents of bioactive constituents with antimycotic and antimycotoxigenic impact [24,53]. It could also be joined to the synergistic effect of the bioactive molecules in the applied extract [45]. The simulated antibacterial experiment was conducted using ready-to-eat rice samples, classified into two (inoculated set using *B. cereus* and a non-inoculated set) sets. The results reflect the more effective use of the aromatic extract for both two sets and at several storage conditions. This finding is connected to the essential and aromatic components in the applied extract [24,32]. The correlation between fungal inhibition and its citrinin reduction could be explained concerning aromatic content, particularly terpenes and monoterpenes [38,40,54]. However, previously cooked rice trials were carried out to test for cooked rice preservation; for example, chitosan as an antimicrobial against *B. cereus* was recoded as a useful application to control measures in ready-to-eat dishes based on pre-cooked rice [55], and it is recommended that be used together with an adequate storage temperature for the microbial load reduction below the infective dose. The present study presented a new strategy to simulate the natural additives used in the normal preparation.

## 5. Conclusions

The preparation of extract from spice mixture could be more effective and rich in bioactive aromatic or phenolic molecules. Encapsulation of the aromatic constituents of the spice by chitosan nanoparticles could prevent their bioactivity against the breakdown during cooking. Furthermore, water extract of spice is known as phenolic bioactive. The application of mixture extract types in the simulated fungi media represents the aromatic extract’s efficacy in anti-citrinin production. Moreover, extracts in ready-to-eat rice samples were shown by shelf life extension impact for both storage conditions (room temperature and refrigerated). These results recommended the application of encapsulated aromatic extract obtained from ginger:thyme:coriander (1:2:1) as a better treatment for shelf life extension of ready-to-eat rice.

## Figures and Tables

**Table 1:** Identification of Aromatic compounds in the extract of (ginger:thyme: coriander) mixture by GC-MS.

Compound	Rt	%	K.I.	Identification
α-thujene	7.78	1.256 ± 0.002	823	MS & KI
α-pinene	10.6	5.389 ± 0.217	933	MS & KI
Camphene	8.4	2.561 ± 0.184	947	MS, KI &ST
Sabinene	9.2	2.877 ± 0.097	966	MS & KI
β-pinene	9.3	1.059 ± 0.055	975	MS, K.I. &ST
β-myrcene	9.8	1.390 ± 0.086	983	MS & KI
m-cymene	13.3	4.175 ± 0.211	991	MS & KI
Citral	16.9	2.608 ± 0.413	992	MS & KI
Decanal	6.4	2.644 ± 0.034	999	MS & KI
D-limonene	11.2	1.366 ± 0.171	1018	MS, KI &ST
1,8 Cineol	10.2	3.242 ± 0.056	1024	MS & KI
P-cymene	11.1	0.825 ± 0.067	1028	MS &KI
Limonene	8.8	2.441 ± 0.039	1031	MS, K.I. &ST
γ-terpinene	11.8	1.058 ± 0.064	1048	MS, KI &ST
2-Decenal	18.2	0.441 ± 0.041	1066	MS & KI
Decanol	21.1	0.582 ± 0.009	1071	MS & KI
Linalool	13.8	9.576 ± 0.422	1083	MS & KI
Camphor	17.6	4.646 ± 0.069	1118	MS, KI &ST
Borneol	16.2	1.069 ± 0.008	1165	MS & KI
α-terpinol	17.1	1.495 ± 0.105	1170	MS & KI
Thymyl acetate	18.5	9.088 ± 0.344	1234	MS & KI
Geraniol	19.6	6.218 ± 0.288	1240	MS, K.I. &ST
carvacrol	18.7	1.442 ± 0.061	1245	MS & KI
Neral	30.1	1.254 ± 0.073	1251	MS & KI
Dodecenal	23.4	0.642 ± 0.008	1257	MS & KI
Thymol	20.8	13.722 ± 0.674	1290	MS, K.I. &ST
Geranyl acetate	20.6	3.184 ± 0.056	1360	MS, K.I. &ST
α-curcumene	23.6	2.066 ± 0.043	1485	MS & KI
α-zingiberene	24.1	6.445 ± 0.088	1496	MS, KI &ST
Elemol	25.6	1.803 ± 0.067	1540	MS & KI
Caryophyllene	24.6	1.784 ± 0.009	1573	MS & KI
t-Farnesol	22.7	1.071 ± 0.005	1725	MS & K.I.

The results are expressed as mean percentage ± SEM (standard error mean; n = 3). Rt: retention time; K.I.: Kovats index for volatile compound identification; MS: mass structure; S.T.: standard internal adding.

**Table 2:** Antiaflatoxigenic properties of applied extracts (water and aromatic) in simulated media.

		A. *flavus* Media		
		G1	G2	G3
Mycelia weight (g)		5.417 ± 0.372	2.058 ± 0.614	2.622 ± 0.218
Reduction ratio (%)		-	62.01%	51.59%
Secreted aflatoxin (ng/mL media)	AFB_1_	219.33 ± 4.31	67.41 ± 5.19	125.23 ± 5.71
	AFB_2_	121.73 ± 3.64	38.16 ± 3.12	71.82 ± 3.14
	AFG_1_	165.21 ± 5.66	58.44 ± 3.54	92.51 ± 2.91
	AFG_2_	81.66 ± 3.17	29.31 ± 2.11	46.56 ± 2.05
Total AFs		587.86 ± 16.78	193.32 ± 13.96	336.12 ± 13.81
Reduction (%)		-	67.11%	42.82%

The results were expressed as means ± SD (n = 3); The aromatic extract was applied in the nano-form of chitosan nanoparticles. *A. flavus* strain is known to produce aflatoxins; WA: water extract; ArE: aromatic extract. G1: flasks contain *A. flavus*; G2: flasks contain *A. flavus* + the W.A.; G3: flasks contain *A. flavus* + ArE.

**Table 3:** Identification of phenolic and flavonoid contents of water extract for the (ginger:thyme:coriander) mixture by HPLC.

Phenolic Acids	Concentrations (mg/100 g)	Flavonoids Compounds	Concentrations (mg/100 g)
Gallic acid	18.41± 3.26	Catechin	13.21 ± 1.21
Pyrogallol	188.8 ± 3.54	Apigenin 7 glucoside	88.3 ± 3.55
Chlorogenic acid	48.63 ± 4.79	Catechol	51.14± 2.81
Protocatechuic acid	15.17 ± 2.37	Epicatechin	29.11 ± 2.54
Trans-ferulic acid	79.84 ± 3.19	Rutin trihydrate	45.73± 1.55
Caffeine	218.4 ± 3.51	Naringenin	645.3 ± 9.41
Vanillic acid	84.22 ± 2.99	Quercetin	202.6± 4.88
Caffeic acid	137.58 ± 4.18	Luteolin 7 glucoside	1448.9 ± 21.7
Ferulic acid	225.71 ± 3.79	Hesperidin	8599.6 ± 28.4
*p*-hydroxybenzoic acid	242.8 ± 6.25	Naringenin-7-o-glucoside	97.36 ± 2.31
Ellagic	456.74 ± 8.64	Rosmarinic	29.7 ± 3.41
Benzoic acid	131.4 ± 3.54	Quercitrin	24.73 ± 4.18
*p*-Coumaric acid	87.9 ± 3.75	Kaempferol 3.7 dirhamoside	228.16 ± 3.79
Salicylic acid	907.16 ± 8.21	Acacetin	69.41 ± 3.16
Coumarin	31.5 ± 3.05	Hispertin	109.3 ± 4.22
Syringic acid	7.87± 2.19	Isorhamnetin-3-o-rutinoside	ND
Cinnamic acid	63.1 ± 2.31	Apigenin	29.9 ± 2.14
Sinapic acid	ND	Chrysin	ND

The ginger:thyme:coriander mixture was applied at a ratio of 1:2:1. The concentrations of the measured compound were represented in mean ± SEM (standard error mean; n = 3). Bioactive ingredients of water extract belonged to phenolic acids and flavonoids; ND: not detected value for the determined compound.

## Data Availability

Data is contained within the article.
